# Detection of copy number variations in brown and white layers based on genotyping panels with different densities

**DOI:** 10.1186/s12711-018-0428-4

**Published:** 2018-11-06

**Authors:** Wioleta Drobik-Czwarno, Anna Wolc, Janet E. Fulton, Jack C. M. Dekkers

**Affiliations:** 1 Department of Animal Science, Iowa State University, 806 Stange Road, 239E Kildee Hall, Ames, IA 50010 USA; 2 Department of Animal Genetics and Breeding, Faculty of Animal Science, Warsaw University of Life Sciences, Ciszewskiego 8, 02-786 Warsaw, Poland; 3 Hy-Line International, 2583 240th Street, Dallas Center, IA 50063 USA

## Abstract

**Background:**

Copy number variations (CNV) are an important source of genetic variation that has gained increasing attention over the last couple of years. In this study, we performed CNV detection and functional analysis for 18,719 individuals from four pure lines and one commercial cross of layer chickens. Samples were genotyped on four single nucleotide polymorphism (SNP) genotyping platforms, i.e. the Illumina 42K, Affymetrix 600K, and two different customized Affymetrix 50K chips. CNV recovered from the Affymetrix chips were identified by using the Axiom^®^ CNV Summary Tools and PennCNV software and those from the Illumina chip were identified by using the cnvPartition in the Genome Studio software.

**Results:**

The mean number of CNV per individual varied from 0.50 to 4.87 according to line or cross and size of the SNP genotyping set. The length of the detected CNV across all datasets ranged from 1.2 kb to 3.2 Mb. The number of duplications exceeded the number of deletions for most lines. Between the lines, there were considerable differences in the number of detected CNV and their distribution. Most of the detected CNV had a low frequency, but 19 CNV were identified with a frequency higher than 5% in birds that were genotyped on the 600K panel, with the most common CNV being detected in 734 birds from three lines.

**Conclusions:**

Commonly used SNP genotyping platforms can be used to detect segregating CNV in chicken layer lines. The sample sizes for this study enabled a detailed characterization of the CNV landscape within commercially relevant lines. The size of the SNP panel used affected detection efficiency, with more CNV detected per individual on the higher density 600K panel. In spite of the high level of inter-individual diversity and a large number of CNV observed within individuals, we were able to detect 19 frequent CNV, of which, 57.9% overlapped with annotated genes and 89% overlapped with known quantitative trait loci.

**Electronic supplementary material:**

The online version of this article (10.1186/s12711-018-0428-4) contains supplementary material, which is available to authorized users.

## Background

Copy number variations (CNV) refer to large-scale insertions, duplications, or deletions of DNA sequence segments compared to a reference assembly. CNV can range in size from 50 to millions of base pairs, but 1 kb is generally assumed to be the lower limit [1, 2]. Most genome-wide mapping studies of CNV have been conducted in humans, where CNV account for a significant proportion of genome variation and are associated with susceptibility to disease [2–5]. According to Zarrei et al. [6], 4.8 to 9.5% of the human genome consists of CNV, while other studies in human and mouse found that CNV explained 18 to 30% of the genetic variation in gene expression [7, 8].

A better understanding of CNV in domesticated animal genomes will contribute to greater genetic improvement of production traits and animal health [9]. Several species of farm animals have been scanned for CNV, including cattle [10–12], sheep [13, 14], goats [15], and pigs [16–18] and numerous studies have examined CNV in the chicken genome [19–32]. Currently, the known CNV in chicken encompass approximately 8.3% of its genome, or 9.6% of the ordered genome assembly [33]. A number of other avian species have also been scanned for CNV, including duck [34] and turkey [19]. Skinner et al. [20] have analyzed CNV in 16 species of birds and found that the number of CNV per Mb was similar in birds and mammals but that their size was smaller in birds than in mammals. In addition, overlapping between CNV and genes in chicken seems to be at the higher end of the range observed in mammals [20], which suggests that CNV may have functional effects in chickens.

According to studies on the human genome, formation of CNV can be connected to differences in recombination rate across the genome [35, 36]. Based on this hypothesis, recombination hot spots should have a higher prevalence of CNV than other parts of the genome. Indeed, based on an analysis of the genomes of chicken and zebra finch, Völker et al. [21] found a significant association between presence of structural variations such as chromosomal rearrangements and recombination rate. These data suggest a major role of recombination-based processes in the evolution of avian genomes.

Copy number variations can be detected by a number of methods, including array comparative genome hybridization (aCGH), sequencing, and single nucleotide polymorphism (SNP) arrays [33]. Although SNP arrays are designed primarily for SNP genotyping, detection of CNV is possible because of the abnormal hybridization that occurs when a SNP is located within a CNV region. In general, the use of a 60K SNP [37] chip for this purpose has resulted in low frequencies of detected CNV [22, 29]. The use of sequence data is much more efficient and yields the largest number of CNV detected [24, 26, 27], but using a 600K SNP array can increase the sensitivity of CNV detection significantly [28]. Four CNV detection studies based on the high-density Affymetrix 600K SNP array have been reported in chicken [28, 30–32] and have shown that, in general, CNV detection with this panel is more efficient than with lower density SNP chips.

Our study aimed at (1) detecting CNV and refining the genome-wide copy number profiles for layer chickens; (2) comparing CNV detection across different SNP genotyping panels, in order to evaluate the utility of these panels for CNV detection; (3) characterizing in detail the differences in CNV detection rates between individuals and lines; and (4) assessing the frequency of detected CNV and their possible functional impact. To achieve this, genes and quantitative trait loci (QTL) that overlap with the detected CNV were identified. Gene enrichment analysis was performed to identify overrepresented biological processes and pathways.

## Methods

### Samples and DNA extraction

The total number of individuals used in this study was 18,719, which included birds from four pure lines, two white shell (W) and two brown shell (B) lines, and from one commercial hybrid of white shell layer chickens, all provided by Hy-Line International (Table 1). DNA was isolated from blood collected from the wing vein of each bird. For the pure lines, blood was collected in EDTA-coated anticoagulant tubes and genomic DNA was extracted following lysis of cells and subsequent digestion with proteinase K. For the commercial hybrids, blood was collected on FTA Elute cards (GE Healthcare) and DNA was extracted following the manufacturer’s recommendations.

**Table 1:** Summary information for the SNP genotyping panels used

Panel	Number of genotyped individuals	Number of genotyped lines	Number of autosomal markers used
42K Illumina panel	1797	1	33,689
50K Affymetrix panel for white layers	6565	2	55,363
50K Affymetrix panel for brown layers	8309	2	54,839
600K Affymetrix panel	2048	4	591,782

### SNP arrays and genotyping

SNP genotypes were obtained from several platforms over multiple years and for multiple purposes (Table 1).

The SNP panels used included the publicly available 600K Affymetrix chip [38], a 42K proprietary Illumina iSelecta BeadChip [39], and two custom 50K Affymetrix chips, which were designed separately for white and brown lines by HyLine International. The choice of SNPs for inclusion in each panel was based on their uniform distribution across the genome. The 42K Illumina panel was optimized to capture the genetic variance that is associated with economically important traits, and thus contained more SNPs in close proximity of genes than the other panels. For both 50K panels, only SNPs with high-quality clusters according to the Axiom™ Analysis Suite were included. This could have led to the elimination of SNPs that overlapped CNV in the birds used for panel design since those SNPs may not form three discrete clusters when plotting allele-A intensity versus allele-B intensity in Axiom™ Analysis Suite.

### Detection of CNV

The Axiom™ Analysis Suite [40] was used to call genotypes for the 50K and 600K Affymetrix panels. A minimum default quality control of 0.82 and a minimum call rate of 97% were used. The Axiom^®^ CNV Summary Tools software [40] was used to extract log R Ratio (LRR) and B allele frequency (BAF) values for PennCNV 1.0.3 [41]. Genotype and CNV calling were performed separately for each 96-well genotyping plate because of the large differences in signal intensities between plates. Data from the 42K Illumina panel were processed in Genome Studio 2011.1 using the Genotyping module v 1.9 and the cnvPartition CNV Analysis Plugin v3.2.0 [42].

PennCNV, an integrated hidden Markov model (HMM), was used for all Affymetrix panels. This algorithm incorporates multiple sources of information, including the signal intensity data of LRR and BAF values at each SNP, the distance between neighboring SNPs, and the population frequency of the B allele (PFB). Individual-based CNV calling was performed using the—test option with default parameters for the HMM model. The hhall.hmm file was used with the—test option for all panels. To adjust for genomic waves, the—gcmodel option with the chicken GC content file (GC content of 1-Mb genomic regions surrounding each SNP) was used. The PFB files were compiled separately for each panel from a large set of individuals, using the compile pfb script included in the PennCNV software. For filtering, standard deviations (SD) for LRR ≤ 0.35, BAF drift < 0.01, and waviness factor ≤ 0.04 were used. The waviness factor accounts for the dispersion in signal intensity across the genome. Only CNV that consisted of at least three (for the 50K and 42K panels) or at least five (for the 600K panel) consecutive SNPs were included in the analysis. Individuals with more than 30 called CNV were excluded as unreliable (58 on the 600K, 47 on the 50K brown, 7 on the 50K white and 33 on the 42K panels). This number (30) was chosen as approximation of the mean number of CNV per individual plus 3 standard deviations across all panels. CNV were identified on autosomes (1 to 28) only because PennCNV calls for the sex chromosomes were unreliable and difficult to interpret.

### Determination of CNV regions

The identified CNV were merged and/or intersected with the BedTools software [43], which combines CNV that overlap in one or multiple interval files into a single CNV region. The BedTools intersect tool was used to select only the region of a CNV that is common between individuals, i.e., if a CNV was identified between 1 and 3 kb in individual 1 and between 2 and 4 kb in individual 2, only the region between 2 and 3 kb was retained in the sets and was referred to as a common CNV region (CNVR). The subsequent sets of CNVR used in this study were obtained as follows:

- For the list of all detected CNVR, a Bedtools merge was performed across all individuals and lines for all CNV that overlapped by at least 1 bp. This set is referred to as the merged CNVR.
- Bedtools merge was performed for variants that were present in at least two individuals within a line. This set is referred to as the common CNVR.
- Bedtools intersect was used for all CNV for a given panel and line combination, which were then merged across panels and lines. CNVR that were identified in at least two individuals within a line, were selected for further analysis. This set is referred to as common intersected CNVR.

CNVR that were detected in only one individual are referred to as singletons. CNVR for which both deletions and duplications were observed are referred to as complex CNVR.

### Annotation of CNVR and gene ontology analysis

Genes that overlapped with common intersected CNVR were identified with the Ensembl BioMart webtool based on the Galgal4 assembly and the Ensembl Genes 85 database [44]. Analysis of overrepresented GO terms and pathways was performed using PANTHER Classification System version 11 [45]. Known quantitative trait loci (QTL) that overlapped with the detected CNVR were identified based on the Animal QTL database [46] release 33. In order to perform a comparison with previous studies, autosomal coordinates of the CNVR were migrated from galGal3 to galGal4 using the UCSC liftOver tool [47]. Common CNV were checked visually using available sequence data, which consisted of representative pools of 10 individuals per line. Details on the sequencing of these pools are in Kranis et al. [38].

## Results

### Detection of CNV

The proportion of samples that passed quality control ranged from 89.4% in line B1 genotyped on the 50K panel up to 99.6% in line W1 genotyped on the 600K panel. For the latter, only one individual was excluded because of poor quality. The mean number of CNV per individual ranged from 0.50 on the 50K panel for line W2 to 4.87 on the 600K panel for line B1 (Table 2). The commercial hybrid cross and line B1 had the largest average number of identified CNV per individual, whereas line W2 had the smallest average number of CNV per individual.

**Table 2:** Summary of CNV identified for each line and each genotyping panel

Line	SNP panel^a^	Number of individuals	Number of individuals that pass quality control	Total number of CNV^b^	Mean N of CNV per individual	Mean length (kb)	Length range (kb)
W1	50K w	3350	3308	2053	0.62	88.1	1.8–955.7
	600K	253	252	772	3.06	25.9	1.2–271.9
W2	50K w	3215	3172	1575	0.50	76.9	1.9–1294.6
	50K b	2401	2253	1844	0.82	111.3	1.4–1493.0
	600K	748	714	1409	1.97	37.2	1.5–428.7
Hybrids (w)	600K	806	769	2261	2.94	31.1	1.4–1116.2
B1	50K b	5908	5284	6203	1.17	216.0	1.7–3160.2
	600K	241	238	1158	4.87	24.9	1.2–663.1
B2	42K	1797	1716	2250	1.31	90.9	1.4–1658.3
All lines		18,719	17,706	19,525	1.10	51.1	1.1–3160.2

^a^*w* white-egg lines, *b* brown-egg lines

^b^Total number of detected CNV for all individuals: all occurrences of CNV were counted

The length of the CNV ranged from 1.2 kb to 3.2 Mb (Table 2). CNV shorter than 1 kb and that included less than 3, 3 or 5 SNPs for the 42K, 50K and 600K panels, respectively, were excluded from analysis. The mean length of detected CNV was greater for the 50K panels, which most likely resulted from the low detectability of shorter variants due to the greater distance between SNPs compared to the 600K panel. The length of each CNV was calculated as the distance from the first to the last SNP included in the CNV region, which may, therefore, slightly underestimate the true length.

Compared to the high-density 600K panel, the 50K SNP panels resulted in a smaller average number of detected CNV per individual (Table 2) and a smaller number of CNVR with a frequency higher than 1% within a line (Table 3). The highest average frequency of CNV detected from the lower density panels was observed for line B2 and the 42K SNP Illumina panel, for which few CNVR had a frequency higher than 1% and one CNV had a frequency higher than 10% (13.9%). The largest number of CNVR with a frequency higher than 1% was observed for the 600K panel (Table 3). A list of the 19 CNVR with a frequency higher than 5% for the 600K panel is in Additional file 1: Table S1. The most common CNVR was detected in 734 individuals across three lines, on chromosome 5 between 19.60 and 19.72 Mb.

**Table 3:** Summary of CNVR detected for each line and genotyping panel

Line	SNP panel^a^	Number of CNVR	Number of deletions		Number of duplications		Number of complex CNVR^c^	Number of CNVR with a frequency > 1%^d^ (maximum)
			Singletons	N ≥ 2^b^	Singletons	N ≥ 2^b^		
W1	50K w	625	89	85	286	98	67	5 (5.7)
	600K	251	101	44	86	12	8	41 (52.8)
W2	50K w	562	60	43	265	139	55	2 (1.2)
	50K b	576	57	40	243	176	60	10 (2.2)
	600K	586	128	56	331	50	21	13 (46.4)
Hybrids	600K	1218	79	30	933	150	25	29 (35.1)
B1	50K b	1146	92	199	419	230	284	17 (2.8)
	600K	440	254	110	50	12	10	73 (33.6)
B2	42K	569	167	95	128	79	100	19 (13.9)

^a^*w* white-egg lines, *b* brown-egg lines

^b^N ≥ 2 = CNVR observed in at least two individuals

^c^Complex CNVR = CNVR within which both deletions and duplications were observed

^d^CNVR with a frequency higher than 1% within line and panel, calculated as the number of individuals with the CNVR divided by number of individuals genotyped that passed quality control (see Table 2 column 4)

**Fig. 1:**
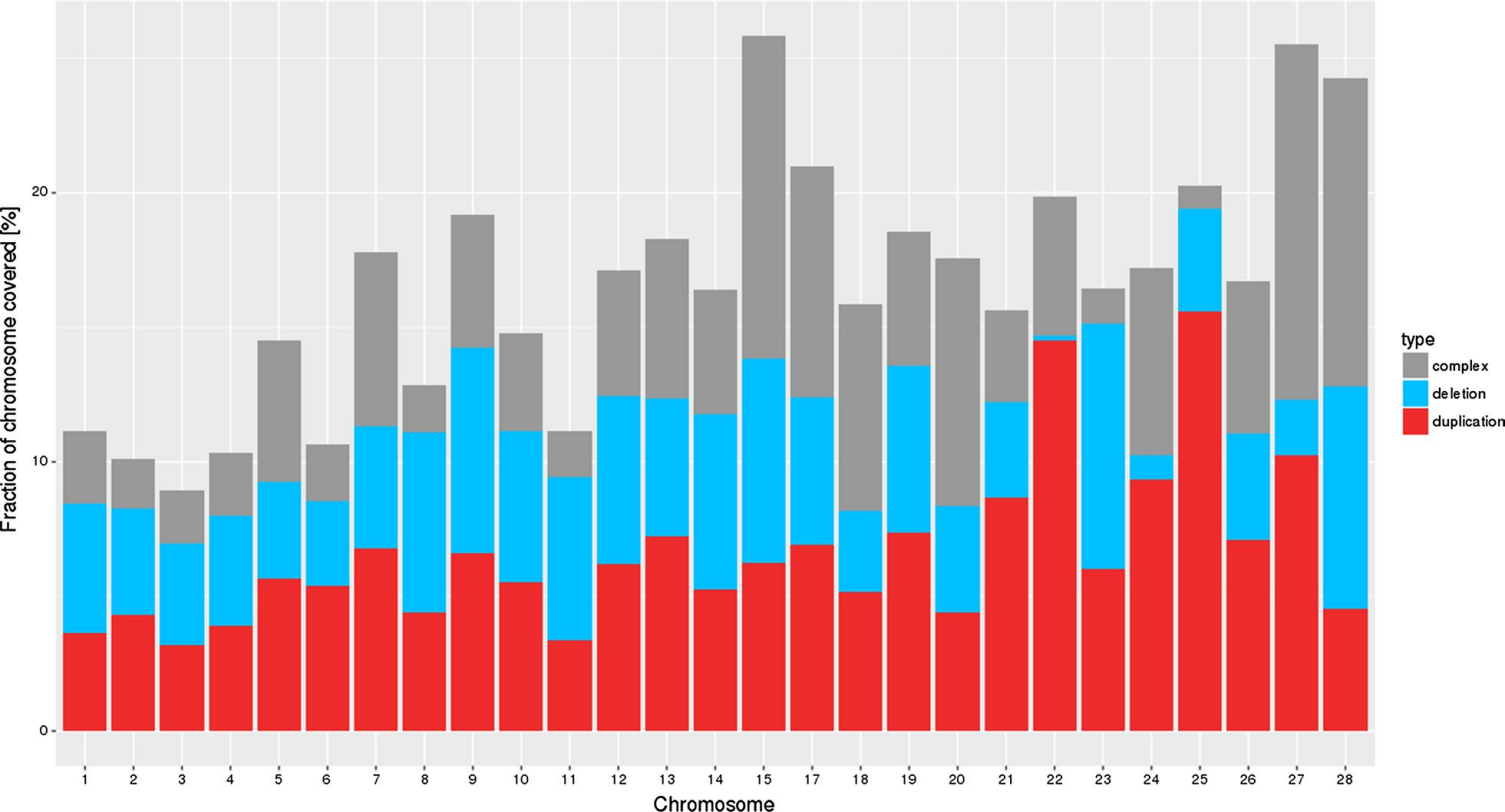
Fraction of each chromosome covered with deletions, duplications and complex CNVR for 2139 common variants

The number of deletions and duplications detected differed between lines (Table 3). The number of duplications exceeded the number of deletions for line W2 and the commercial hybrid, while for line B2 more deletions were detected. For lines W1 and B1, the ratio of duplications to deletions differed between panels. In some regions, complex CNVR were observed, but the number of complex CNVR identified was significantly larger for the lower density panels than for the 600K panel (Table 3), which probably resulted from the poorer variant separation with the lower density panels. The proportion of each chromosome that was covered with deletions, duplications, or complex variants for common CNVR is shown in Fig. 1. The fraction covered with complex CNVR was greater for the microchromosomes (6% on average) than for the macrochromosomes (on average 3% of the total sequence). Although the number of duplications exceeded the number of deletions for most lines, both types of variants covered a similar fraction of the chromosomes.

### CNVR and overlapping genes

The distribution of CNVR differed between chromosomes, with microchromosomes having a higher density of CNVR than macrochromosomes (Table 4). The log10 of chromosome size was inversely correlated with the fraction of chromosome covered by CNVR, with a Pearson correlation of − 0.80. Chromosome 16 is the shortest chromosome in the chicken genome with a large fraction covered with CNV. However, results for chromosome 16 should be treated with caution, since the reference sequence for this chromosome is of poor quality, probably because it carries the major histocompatibility complex, which has a high level of variability, multiple gene families, and a high GC content.

**Table 4:** Summary of CNVR per chromosome for all panels

Chr	Chr length (Mb)	Merged CNVR^a^	Merged CNVR N ≥ 2^b^		Intersected CNVR N ≥ 2^c^		
		Number	Number	Fraction of chr covered^d^	Number	Fraction of chr covered^d^	Genes^e^
1	195.3	544	234	0.370	378	0.111	0.050
2	148.8	435	186	0.322	264	0.101	0.036
3	110.4	317	138	0.321	192	0.089	0.036
4	90.2	258	113	0.389	172	0.103	0.042
5	59.6	184	85	0.394	136	0.145	0.076
6	35.0	135	59	0.362	87	0.107	0.064
7	36.2	86	46	0.522	96	0.178	0.100
8	28.8	79	38	0.429	68	0.128	0.061
9	23.4	63	37	0.497	67	0.192	0.091
10	19.9	72	38	0.452	63	0.148	0.078
11	19.4	55	27	0.432	51	0.112	0.061
12	19.9	54	27	0.528	58	0.171	0.111
13	17.7	61	26	0.480	43	0.183	0.084
14	15.1	46	26	0.457	47	0.164	0.094
15	12.7	22	18	0.760	55	0.258	0.141
16	0.5	1	1	0.979	2	0.923	0.564
17	10.4	26	16	0.677	44	0.210	0.102
18	11.2	30	18	0.523	39	0.158	0.084
19	10.0	17	12	0.786	40	0.186	0.127
20	14.3	43	20	0.495	39	0.175	0.085
21	6.8	35	17	0.456	30	0.156	0.078
22	4.1	8	5	0.664	17	0.199	0.068
23	5.7	21	13	0.593	28	0.164	0.091
24	6.3	24	12	0.626	28	0.172	0.111
25	2.2	4	5	0.701	16	0.203	0.092
26	5.3	25	16	0.485	22	0.167	0.083
27	5.2	24	18	0.698	27	0.255	0.183
28	4.7	18	13	0.622	30	0.243	0.143

*Chr* chromosome

^a^Merged CNVR = All CNVR merged across all lines; 2687 CNVR in total

^b^Common CNVR N ≥ 2 = Merged CNVR observed in at least two individuals within a line, merged across all lines; 1264 CNVR in total

^c^Common intersected CNVR N ≥ 2 = Intersected CNVR observed in at least two individuals within a line, merged across all lines; 2139 CNVRs in total

^d^Fraction of chromosome covered with CNVR

^e^Fraction of CNVR overlapping with genes

In total, 2687 CNVR were identified after merging CNV across all samples and lines (Additional file 2: Table S2). The total length of these CNV was equal to 493.3 Mb, which corresponds to 53.7% of the analyzed genome sequence. Of the merged CNVR, 73.4% overlapped with genes, which accounted for 45.9% of the total CNVR sequence.

The number of common CNVR, which resulted from merging CNV that were found to be shared by at least two individuals within a line, was equal to 1264, with a total length of 375.8 Mb. Of these CNVR, 82.0% overlapped with genes, which encompassed 46.2% of the total CNVR sequence. More than 97.2% of the merged CNVR and 96.8% of the common CNVR overlapped with known QTL. Of the 1264 CNVR, 447 CNVR that cover 252.8 Mb, were detected by more than one SNP panel.

Intersecting CNVR across all lines and panels resulted in 4131 CNVR. Since the CNV that were observed once require further confirmation, CNVR, which were identified in at least two individuals within a line after intersecting within panels, were selected for further analysis (N = 2139). The total length of these common intersected CNVR was equal to 117.3 Mb, which corresponds to 12.7% of the genome. In total, 29.8% of these CNVR overlapped with 3510 Ensembl gene ID, for which 2322 gene names were available, including 94 miRNAs and 29 LOC genes (Additional file 3: Table S3). Of the 3510 Ensembl gene ID, 2994 genes mapped to Panther biological categories. GO enrichment analysis of these genes revealed significant terms involved in antigen processing and presentation, and cellular defense response, which may represent biological processes that are influenced by CNV (Table 5).

**Table 5:** Gene ontology (GO) overrepresented terms for common CNVR (N = 2139)

PANTHER GO-Slim biological process	GO term	REFLIST (15,696)	Input (2994)	Expected	Fold enrichment	p-value
Antigen processing and presentation	GO:0019882	39	22	7.44	2.96	1.10E−05*
B cell mediated immunity	GO:0019724	80	29	15.26	1.90	1.08E−03
Fatty acid biosynthetic process	GO:0006633	31	11	5.91	1.86	3.90E−02
Cellular defense response	GO:0006968	165	58	31.47	1.84	1.29E−05*
Chromosome segregation	GO:0007059	75	24	14.31	1.68	1.16E−02
Synaptic vesicle exocytosis	GO:0016079	62	19	11.83	1.61	3.29E−02
Chromatin organization	GO:0006325	165	47	31.47	1.49	5.52E−03

The terms that are significant after Bonferroni correction are marked with *

### Within-line CNV characterization

Each line was characterized by its own CNV profile. Since only the high-density 600K panel was used for more than two lines, the comparison of CNV profiles between lines was based on this panel only. The number of CNVR that were common between lines is in Table 6. The largest overlap was observed between line W2 and the commercial hybrid line, which may be related to the fact that these lines had the largest number of individuals and CNV detected. As expected, line B1 had a relatively small number of common CNV with the white lines and the hybrid cross, which can be explained by the relatively large genetic distance between white and brown egg shell lines. This difference was most pronounced with line W1, for which the number of genotyped individuals was smallest.

**Table 6:** Number of CNVR that overlapped between lines (above diagonal)

Line	Hybrids	W1	W2	B1
Hybrids	1218	55	123	46
W1	2.88 (42.5)	251	24	13
W2	9.51 (45.1)	10.14 (69.0)	586	33
B1	6.15 (55.1)	2.56 (30.3)	7.34 (53.3)	440

The total number of CNVR within a line is on the diagonal and the % of Mb coverage for all CNVR is under the diagonal (% of Mb coverage for overlapping CNVR in brackets)

To determine whether CNV are associated with specific biological processes, we identified the genes within CNVR that were detected in at least two individuals within a line. In total, 682 genes overlapped with 465 merged CNVR for the 600K panel and 602 of these were mapped by Panther with 257 CNVR not classified in any GO term. Two terms were significant after Bonferroni correction, phagocytosis (p-value = 0.0250) and cellular defense response (p-value = 0.0127), with enrichments of 5.71 and 3.25, respectively.

Then, we performed the Panther GO overrepresentation test for genes that were identified within each line separately. No significant GO terms were identified for line W1, probably because of the small number of genotyped individuals and the small number of detected CNVR. For all the other lines, we detected several significant GO terms, but these were mostly connected to genes that overlapped with a single CNVR. For the hybrid cross, the most significant GO terms were: antigen processing and presentation, phagocytosis, B cell immunity, and cellular defense response. The antigen processing and presentation term was connected to CNVR that were identified on chromosome 16, which consisted of 13 CNV that covered almost the entire chromosome. The B cell immunity and cellular defense response terms, which were significant for lines B1, W2, and the hybrid cross, were connected to a region on chromosome 27, where a single copy deletion was observed between 0.19 and 0.33 Mb (Table 2).

### Confirmation of CNVR with frequencies higher than 5%

**Fig. 2:**
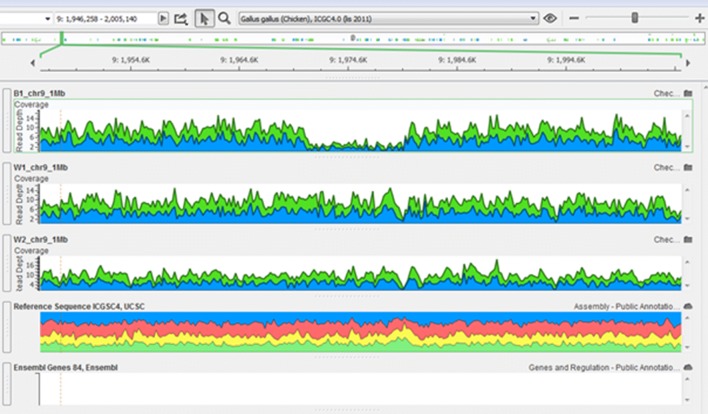
CNV on chromosome 9 (between 1.968 and 1.978 Mb) within line B1

Information about the confirmation of CNVR based on sequence data of pooled DNA is provided in Additional file 1: Table S1. Due to lack of individual sequence data, only CNV with a relatively high frequency could be detected based on sequence information. In addition, because of the small number of individuals in each pool of sequenced data, even relatively frequent CNV may be indistinguishable from noise. Examples of CNV that were confirmed by sequence data are in Fig. 2 and in Additional file 4: Figures S1 to S8. The sequence data enabled confirmation of selected variants but did not provide a means for identifying false positives because sequenced individuals represented only a limited number of individuals of the genotyped lines and no individual had both sequence and SNP genotype data.

## Discussion

In this study, we used 17,706 individuals from four pure lines and one commercial multi-line cross to detect CNV using genotypes provided by four SNP panels with different densities. In total, 19,525 CNV were detected, which resulted in 2687 CNVR after merging across individuals, lines, and panels. This result shows that CNV detection is possible by using commercially available SNP genotyping platforms. In addition, 19 high frequency CNVR were detected using the 600K panel, of which 57.9% overlapped with annotated genes (Additional file 1). We hypothesize that the CNV, which segregate within the lines at relatively high frequencies, may have an impact on the traits that are under selection in these lines.

### CNV detection and comparison of results between SNP panels

Similar to other studies on the detection of CNV, duplications were more abundant than deletions [22, 30], although there were some differences between lines. For line B2, the number of losses was almost equal to the number of gains, which may be specific of this line or of the 42K panel, which was initially developed to exclude SNPs that did not perform well (thus some SNPs within CNV may have been eliminated). For line B1, the 600K panel, the number of losses was almost six times larger than the number of gains when the 600K panel was used, whereas interestingly, when the 50K panel was used, we obtained the opposite result, although most of the gains were due to singletons. These results may be due to the large difference in the number of line B1 individuals genotyped for these two panels, the low detectability with the 50K panel, and the large number of singletons.

Based on the literature, generally most of the detected CNV have low population frequencies, although the use of relatively small numbers of individuals can result in sampling bias. According to Jia et al. [22], among the 315 CNV that they detected in an analysis of 746 chickens with the 60K SNP array, only four had a frequency higher than 5% and none had a frequency higher than 10%. In addition, more recent studies have reported that most of the detected CNVR are singletons (occurring only in one individual), i.e. 76% in Han et al. [25], 69% in Yi et al. [26], and 75% in Strillacci et al. [31]. In our study, we detected several common CNVR with a frequency higher than 5%, although most of these were detected only with the 600K panel (Table 2). The lower frequency of the CNVR detected when using the 50K and 42K panels confirm the advantage of higher SNP densities for CNV detection as previously reported [28, 30, 31].

Among all the detected CNVR, 46% were observed in a single individual across all lines. This observation, combined with the large number of individuals used in this study, confirms previous observations that a large fraction of CNV are singletons. However, such a large number of singletons could also result from the stringent quality control criteria that were applied in this study, including plate-by-plate detection, which could result in some CNV being overlooked.

**Fig. 3:**
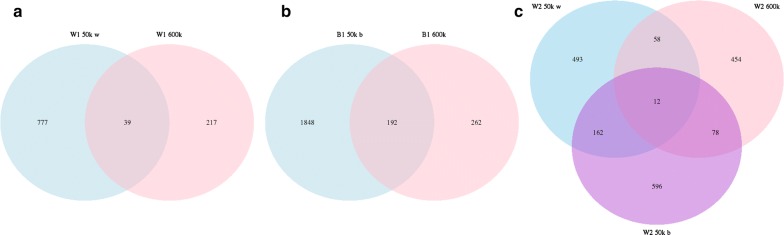
**a**–**c** Comparison of the number of CNVR detected with different SNP panels within pure lines

The number of common CNV (present in at least two individuals within a line) that were detected within and across lines is shown in Fig. 3. The number of CNVR that were shared between the four pure lines and between SNP panels was rather small, which could be due to the relatively small number of CNV per individual that was obtained with the 50K panels and to the large number of singletons. Among the lower density panels, the largest number of CNV per individual was found with the 42K Illumina panel, which may be related to differences in genotyping technology or line specificities.

The stringent quality control criteria that were applied when selecting SNPs for the 50K panel may have excluded SNPs in CNV regions. This hypothesis is supported by the larger number of CNV per individual, which were detected for the white layer line W1 when using the 50K panel that was developed specifically for brown layers compared to the 50K panel that was developed specifically for white layers (Table 1).

To summarize, although all panels enable the detection of CNV, it is possible that a proportion of the CNV that could be detected by more accurate data such as sequence data are missed when using SNP panels, especially lower density panels. In addition, a number of characteristics should be taken into account when calling CNV with SNP panels. First, distance between SNPs on the panel and their coverage have a clear effect on the length of the detected CNV. Second, it is necessary to have genotypes for a relatively large number of individuals to detect CNVR that are segregating within populations and to estimate their frequency. The pre-selection strategy for the SNPs placed on the panel also needs to be taken into account, since the SNPs that are located within CNV are more likely to be excluded as non-performing. Finally, the panel used can influence the ratio of detected deletions to duplications. In light of these results, our recommendation is that CNV detection using SNP genotypes can be used on a larger scale for commercial populations with large sample sizes, but keeping in mind the limitations.

### Chromosome coverage and gene content

The number of CNV detected varied between chromosomes, with the microchromosomes being characterized by a higher density of CNV. In general, microchromosomes are known to have a higher gene content, which directly contradicts the observation that the majority of CNV are in gene-poor regions and gene deserts [28]. Our results suggest the opposite, i.e. that microchromosomes are more CNV-rich than macrochromosomes and thus more frequently associated with genes, which is consistent with the findings of Skinner et al. [20]. One of the most interesting cases is chromosome 16, which was covered at 98% by CNV, these being present in 53 individuals across all lines. This confirms a number of previous studies [28, 30]. The reason for this high density of CNV on chromosome 16 could be that it carries the major histocompatibility complex and has a high recombination rate, but the poor reference genome sequence for this chromosome could also be a cause. Details on the recombination rate and CNV located on chromosome 16 are in Fulton et al. [48].

Rao et al. [29] reported that only 38% of the 383 CNVR that they identified in chickens overlapped with genes. We observed a similarly small percentage (30%) for the 2139 intersected CNVR, which is probably related to the relatively short length of the intersected variants that fall within intergenic regions. These results support the hypothesis that a majority of CNVR is associated with genes and may have functional effects. In contrast, in a study on 16 bird species, Skinner et al. [34] determined that 70% of the detected CNVR overlapped with genes. We obtained a similar result for merged CNV, of which 73.4% overlapped with genes, and this percentage was even higher for the 1264 common CNVR (82.0%). These results support the hypothesis that the majority of CNVR is associated with genes and may have functional effects. In addition, GO analysis showed that genes that overlapped with CNV were enriched with a number of biological functions, in particular related to immune response. This is consistent with the results of Jia et al. [22] who suggested that this type of polymorphism might be prevalent in immune-related genes.

### Comparison of CNVR detected in our work with previous studies

Additional file 5: Table S4 includes the list of the CNVR that were detected in this study and that overlap with previously detected CNVR. Of the 2687 CNVR that we detected, 70% overlapped with previously detected CNV, but these only comprise 28% of the total sequence length for all CNVR detected in this study. Of the 1264 common CNVR, 169 were novel and covered 2.4% of their total sequence (375.8 Mb). The total sequence overlap of common CNVR with previously known CNVR was equal to 32.6%, which can be related to the large length of merged CNVR. For both all and common CNVR, the sequence coverage with previously detected CNVR was around 30%. This observation, combined with the large number of singletons, leads to the conclusion that the occurrence of CNV is specific for each individual and inter-individual differences are more pronounced than between-line differences.

Our results show that the use of the high-density 600K panel greatly improves the detection of CNV compared to that of low-density panels. Four studies have already used this 600K panel to detect CNV in various breeds or lines of chickens, and these are summarized in Table 7 [28, 30–32]. On average, the number of CNV per individual was larger in those studies than in ours, probably because of the higher level of genetic variability in indigenous breeds than in highly selected commercial lines, such as those that we investigated. This was confirmed by Yi et al. [28], who found that the average number of CNV detected in commercial breeds was equal to 3.3 versus 5.1 for Chinese indigenous breeds. The populations used for CNV detection by Gorla et al. [30] and Strillaci et al. [31] were also characterized by higher genetic variability. We detected a larger number of CNV per individual in the brown line B1 (4.87), which is close to what was reported for some non-commercial breeds [28, 30, 31]. In contrast, the smallest number of CNV per individual was detected in line W2 for the 600K panel, which has a relatively high level of inbreeding (results not shown).

**Table 7:** Summary of CNV detected in the chicken genome based on the 600K panel in the current and previous studies

Source	N^a^	Number of breeds or lines	Mean number of CNV per individual	Mean CNV length (kb)	Number of CNV detected	Number of CNVR	Mb covered by CNVR
[31]	96	6	10.7	19.6	1003	564	9.4
[28]	96	12	5.0	27.6	418	231	5.6
[30]	256	Not clear^b^	7.5	38.7	1924	1216	47.0
[32]	30	4	5.6	4.9	–	173	0.8
Current work (600K data only)	2048	4	2.7	30.6	5616	2689	493.3

^a^Number of individuals used in the study

^b^Diverse Mexican chicken population without clear breed classification

Overall, we found a larger total number of CNV and a higher proportion of the genome covered by these CNV than previous studies in chickens [28, 30–32]. These differences are likely due to the much larger number of individuals analyzed in this study (Table 7), which allowed a better characterization of within-line CNV variability.

### High-frequency CNVR

Of the 19 CNVR that were identified (based on the 600K panel) with a frequency of at least 5% within one line, 11 overlapped with at least one gene and 17 overlapped with a previously detected QTL (Additional file 6: Table S5). Among the QTL that overlapped with these common CNVR, 26 were involved in body weight and 13 in growth. The largest numbers of overlapping QTL were found for CNVR on chromosomes 2, 3, 4 and 5. The deletion on chromosome 8 overlapped with the largest number of genes (14) and with one QTL, for body weight. The GO terms that were enriched for the 600K CNV were mostly related to immune-response genes. This observation, along with the large number of singleton CNV identified, suggests a large inter-individual variability among the genes involved in immune response.

The high-frequency CNVR located at 179 Mb on chromosome 1 overlapped with a number of QTL for body weight and Marek’s disease related traits, and with the *ALKBH8* gene. Wang et al. [49] had already reported this CNVR on chromosome 1 between 184,874,498 and 184,879,098 bp in build 3 of the chicken genome and predicted 45 candidate transcription factor binding sites for this region by WWW PROMOTER SCAN. This suggests that amplification of this upstream locus might affect expression of the *ALKBH8* gene, which codes for tRNA methyltransferase and is involved in tRNA modifications and regulation of gene expression.

The second interesting high-frequency CNVR is a single copy deletion on chromosome 6 (12.47–12.54 Mb). This deletion does not overlap with a gene but it is located in close proximity to a number of genes, downstream to *ZMIZ1* and upstream to *RPS24* and *POLR3A*, which are all involved in immune response. This region also overlaps with 10 QTL, including one for antibody response to sheep red blood cells (SRBC).

The high-frequency CNVR deletion on chromosome 23 (between 2.34 and 2.35 Mb) overlaps with the gene *RHCE* (Rh blood group CcEe antigens). Previously detected QTL located within this region are involved in body weight and shank length. A duplication on chromosome 2 between 129.10 and 129.17 Mb overlaps with two genes, *BAALC* and *FZD6*, which are both connected to immune response. A CNV that overlaps with the *FZD6* gene was previously reported by Yan et al. [27] and was associated with Marek’s disease resistance. This CNV also overlaps with two QTL in the Animal QTL database that are related to Marek’s disease and with cloacal bacterial burden following challenge with Salmonella.

## Conclusions

Our results support previous findings that a large proportion of all detected CNVR are singletons, but we were able to detect several common CNVR, which may have important functional impacts. In addition, the large number of CNV that overlap with genes suggests that chicken CNV can impact agricultural or disease-related traits. In this context, the detection of structural variants such as CNV in chicken should be performed on a wider scale. The use of SNP genotypes on a large number of individuals enabled a better characterization of the CNV, both within and between lines. The list of CNVR presented here provides an additional resource for further studies in chicken. We observed pronounced differences between SNP panels and a clear advantage for the dense 600K SNP panel, both regarding the total number of CNV detected and their population frequencies. Although the use of SNP panels does not allow all the CNV that are present in an individual to be detected, these results show that they are a valuable source of CNV information by allowing the screening of large numbers of individuals at relatively low cost.

## Additional files

**Additional file 1: Table S1.** Summary of CNVR with a frequency higher than 5% within at least one line based on the 600K panel. The data provided represent details for 19 CNVR with a frequency higher than 5% detected on the 600K panel. For each CNVR location, line of origin, type, frequency, overlap with the previous studies, confirmation from sequence (yes or no) and overlapping genes are provided.

**Additional file 2: Table S2.** CNVR identified after merging all CNV across samples and lines. The table contains CNVR identified after merging all detected CNV across all samples and lines. For each CNVR coordinates, length, copy number variation type, number of individuals with CNV and panels within which they were observed are provided. CNVR_id is introduced as CNVR identification and is referred to in Additional file 3 Table S3, Additional file 5 Table S4 and Additional file 6 Table S5.

**Additional file 3: Table S3.** Intersected CNVR identified in at least two individuals. The table contains all intersected CNVR identified in at least two individuals within a line after intersecting within panels. For each CNVR coordinates, length and all overlapping genes are provided.

**Additional file 4: Figure S1.** Duplication on chromosome 1 of 179.8 Mb, which overlaps with the gene *ALKBH8* and segregates in line W1. Coverage plot from Golden Helix Genome Browse for CNVR and neighboring regions. **Figure S2.** Duplication on chromosome 2 of 129.1 Mb, which overlaps with the *BAALC* and *FZD6* genes and segregates in line B1. Coverage plot from Golden Helix Genome Browse for CNVR and neighboring regions. **Figure S3.** Complex CNV region on chromosome 4 of 61.8 Mb that segregates in white lines. Coverage plot from Golden Helix Genome Browse for CNVR and neighboring regions. **Figure S4.** Complex CNV region on chromosome 5 of 0.1 Mb that segregates in line B1. Coverage plot from Golden Helix Genome Browse for CNVR and neighboring regions. **Figure S5.** Deletion on chromosome 5 of 19.6 Mb that segregates within the white lines W1 and W2. Coverage plot from Golden Helix Genome Browse for CNVR and neighboring regions. **Figure S6.** Deletion on chromosome 9 of 1.9 Mb that segregates within line B1. Coverage plot from Golden Helix Genome Browse for CNVR and neighboring regions. **Figure S7.** Complex CNV region on chromosome 12 of 2.0 Mb that overlaps with the *DOCK3* gene and segregates within line B1 and W1. The duplication segregates in line B1 while the deletion segregates in line W1. Coverage plot from Golden Helix Genome Browse for CNVR and neighboring regions. **Figure S8.** Deletion on chromosome 23 of 2.5 Mb that overlaps with the *EPB41* gene and segregates within line B1. Coverage plot from Golden Helix Genome Browse for CNVR and neighboring regions.

**Additional file 5: Table S4.** Overlaps with previously detected CNVR. The data provided represents the list of all CNVR detected in this study and their overlaps with previously detected CNVR.

**Additional file 6: Table S5.** Overlaps with detected QTL for high frequency CNVR. The data provided represents overlaps with a previously detected QTL for the 19 CNVR that were identified (based on the 600K panel) with a frequency of at least 5% within one line.
