# Supplementary material for: Detection of copy number variations in brown and white layers based on genotyping panels with different densities
Source: Genet Sel Evol. 2018 Nov 6;50:54. doi: 10.1186/s12711-018-0428-4 (PMC6219011; doi:10.1186/s12711-018-0428-4)
Supplement: Supplementary file 1 — Additional file 1: Table S1. Summary of CNVR with a frequency higher than 5% within at least one line based on the 600K panel. The data provided represent details for 19 CNVR with a frequency higher than 5% detected on the 600K panel. For each CNVR location, line of origin, type, frequency, overlap with the previous studies, confirmation from sequence (yes or no) and overlapping genes are provided. [file 12711_2018_428_MOESM1_ESM.docx]

Table S1. Summary of CNVRs with frequency above 5% within at least one line based on the 600k panel.

| Chr | Start | Stop | Type | Line | Freq [%] | Length [kb] | Previous studies | Other lines | Confirmed | Overlapping genes |
| --- | --- | --- | --- | --- | --- | --- | --- | --- | --- | --- |
| 1 | 179799325 | 179823362 | dup | W2 | 8.3 | 24.1 | Yes [6; 100.0 %] | W1, Hybrids | Yes (seq) | ALKBH8 |
| 2 | 111417623 | 111463419 | del | W1 | 5.1 | 45.8 | novel | B1, W2, Hybrids | Yes (panel) | - |
| 2 | 129100114 | 129172949 | dup | B1 | 24.4 | 72.8 | Yes [9; 100%] | - | yes | BAALC,  FZD6 |
| 2 | 132524192 | 132530162 | complex | W1 | 32.1 | 6.0 | novel | B2 | no | - |
| 3 | 22584687 | 22794542 | complex | B2 | 13.9 | 209.9 | Yes [2; 4.4 %] | B1, Hybrid | not available | ENSGALG00000009912  PKDCCa |
| 3 | 26662612 | 26669313 | del | W1 | 7.1 | 6.7 | Yes [1; 99.8 %] | Hybrid, W2, B1 | yes | PLB1,  gga-mir-6580 |
| 4 | 61822333 | 61842179 | complex | W1 | 22.1 | 19.9 | Yes [6; 100.0 %] | Hybrid, W2, B1, B2 | yes | - |
| 5 | 177005 | 208313 | complex | B1 | 31.0 | 31.3 | Yes [4; 100.0 %] | W1, W2, Hybrid | yes | UNC93B1, ALDH3B1, NDUFS8, TCIRG1, TBX10 |
| 5 | 19602014 | 19724489 | complex | Hybrids  W1  W2 | 33.5  52.6  44.3 | 122.5 | Yes [12; 100.0 %] | All | Yes (seq) | - |
| 6 | 12471871 | 12538088 | del | Hybrids | 10.2 | 66.2 | Yes [8; 100.0 %] | W1 | not available | - |
| 8 | 25023 | 1332836 | del | B1 | 8.7 | 1307.8 | Yes [10; 96.9 %] | Hybrids, B2, W2 | no | 14 genes * |
| 9 | 1968234 | 1978197 | complex | B1 | 28.1 | 10.0 | Yes [1; 63.9%] | - | Yes (seq) | - |
| 10 | 2413818 | 2417564 | del | W1 | 9.1 | 3.7 | Yes [2; 100.0 %] | B1, B2 | no | - |
| 10 | 8693067 | 8715744 | del | B1 | 12.0 | 22.7 | Yes [2; 100.0 %] | B2 | no | - |
| 12 | 2073133 | 2095723 | complex | W1 | 28.1 | 22.6 | Yes [5; 88.0 %] | Hybrid, B1 | yes | DOCK3 |
| 21 | 1878272 | 1884318 | del | B1 | 5.8 | 6.1 | novel | W2, B2 | no | ENSGALG00000001288 |
| 23 | 2343173 | 2351084 | complex | B1 | 28.1 | 7.9 | Yes [5; 100.0 %] | Hybrid, W1, W2 | yes | RHCE |
| 23 | 2568013 | 2575250 | complex | B1 | 33.1 | 7.2 | Yes [2; 100.0 %] | W2 | yes | EPB41 |
| 27 | 209790 | 333683 | complex | W2 | 44.3 | 123.9 | Yes [7; 100.0 %] | Hybrid, B1 | no | ENSGALG00000024248  ENSGALG00000024254 ENSGALG00000027010 ENSGALG00000024255 |

14 genes * - MY2A, NTNG1, ENSGALG00000021244, VAV3, ENSGALG00000021240, SLC25A24, FAM102B, ENSGALG00000027951, HENMT1, PRPF38B, FNDC7, STXBP3, GPSM2, CLCC1

Confirmed - with other panel for line / sequencing

Previous studies - [number of studies; coverage]
