# Supplementary material for: Detection of copy number variations in brown and white layers based on genotyping panels with different densities
Source: Genet Sel Evol. 2018 Nov 6;50:54. doi: 10.1186/s12711-018-0428-4 (PMC6219011; doi:10.1186/s12711-018-0428-4)
Supplement: Supplementary file 4 — Additional file 4: Figure S1. Duplication on chromosome 1 of 179.8 Mb, which overlaps with the gene ALKBH8 and segregates in line W1. Coverage plot from Golden Helix Genome Browse for CNVR and neighboring regions. Figure S2. Duplication on chromosome 2 of 129.1 Mb, which overlaps with the BAALC and FZD6 genes and segregates in line B1. Coverage plot from Golden Helix Genome Browse for CNVR and neighboring regions. Figure S3. Complex CNV region on chromosome 4 of 61.8 Mb that segregates in white lines. Coverage plot from Golden Helix Genome Browse for CNVR and neighboring regions. Figure S4. Complex CNV region on chromosome 5 of 0.1 Mb that segregates in line B1. Coverage plot from Golden Helix Genome Browse for CNVR and neighboring regions. Figure S5. Deletion on chromosome 5 of 19.6 Mb that segregates within the white lines W1 and W2. Coverage plot from Golden Helix Genome Browse for CNVR and neighboring regions. Figure S6. Deletion on chromosome 9 of 1.9 Mb that segregates within line B1. Coverage plot from Golden Helix Genome Browse for CNVR and neighboring regions. Figure S7. Complex CNV region on chromosome 12 of 2.0 Mb that overlaps with the DOCK3 gene and segregates within line B1 and W1. The duplication segregates in line B1 while the deletion segregates in line W1. Coverage plot from Golden Helix Genome Browse for CNVR and neighboring regions. Figure S8. Deletion on chromosome 23 of 2.5 Mb that overlaps with the EPB41 gene and segregates within line B1. Coverage plot from Golden Helix Genome Browse for CNVR and neighboring regions. [file 12711_2018_428_MOESM4_ESM.docx]

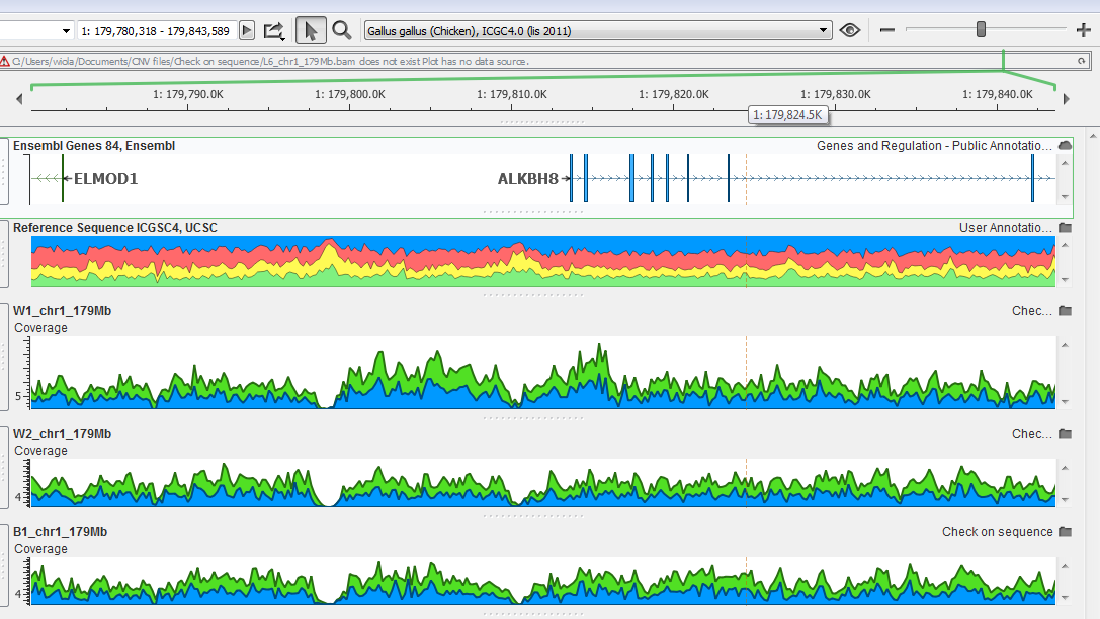


Figure S1. Duplication on chromosome 1 of 179.8 Mb, which overlaps with the gene ALKBH8 and segregates in line W1.


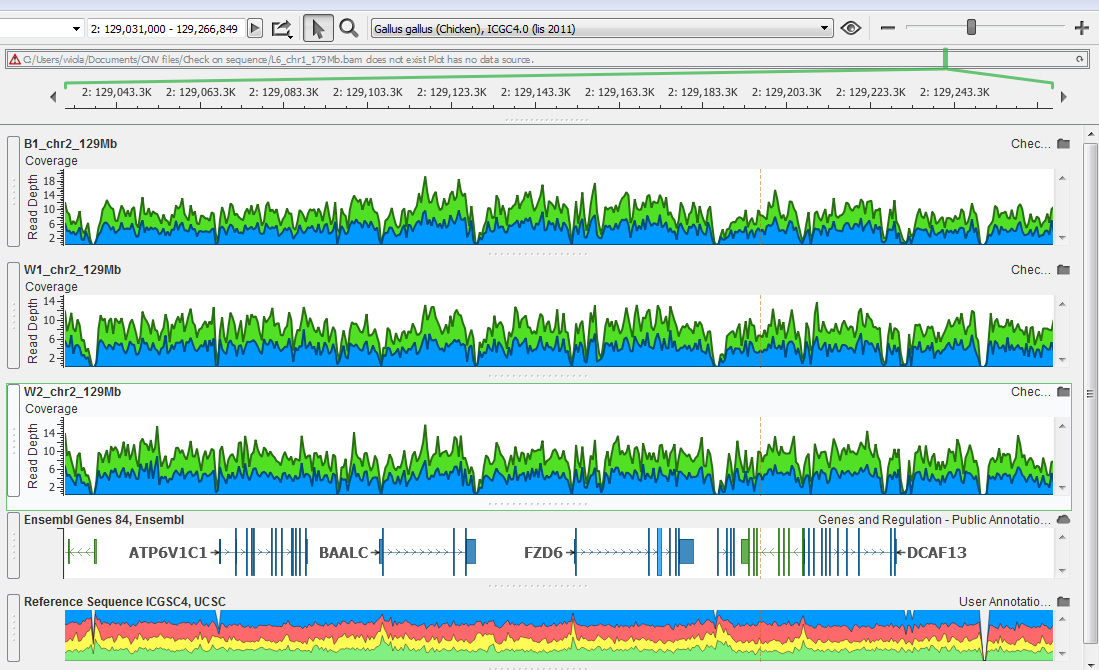


Figure S2. Duplication on chromosome 2 of 129.1 Mb, which overlaps with the BAALC and FZD6 genes and segregates in line B1.


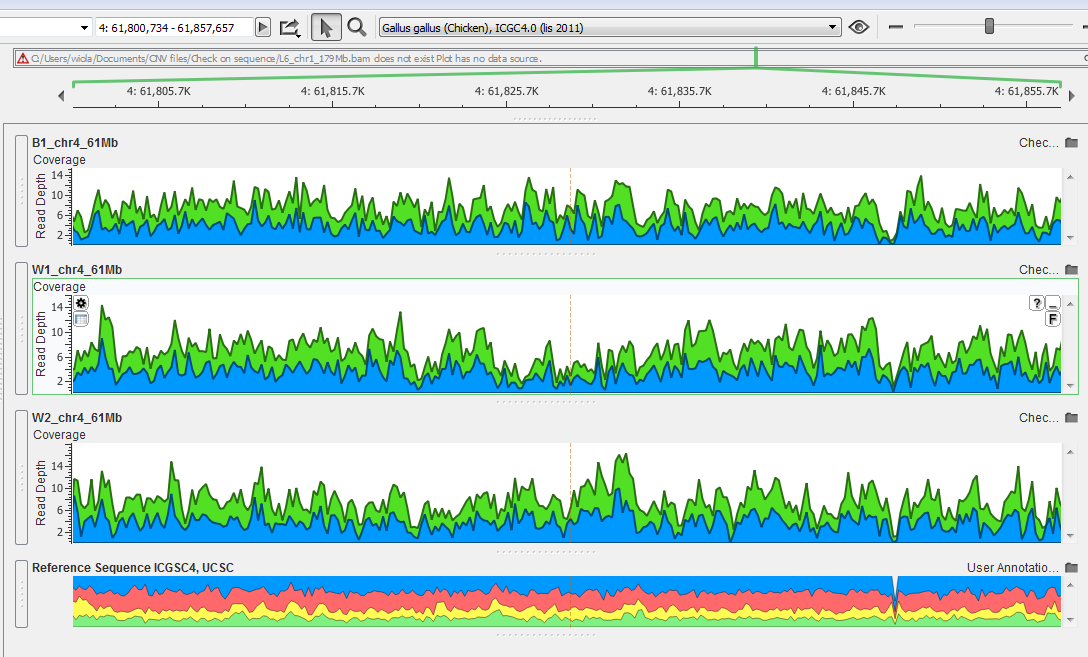


Figure S3. Complex CNV region on chromosome 4 of 61.8 Mb that segregates in white lines.


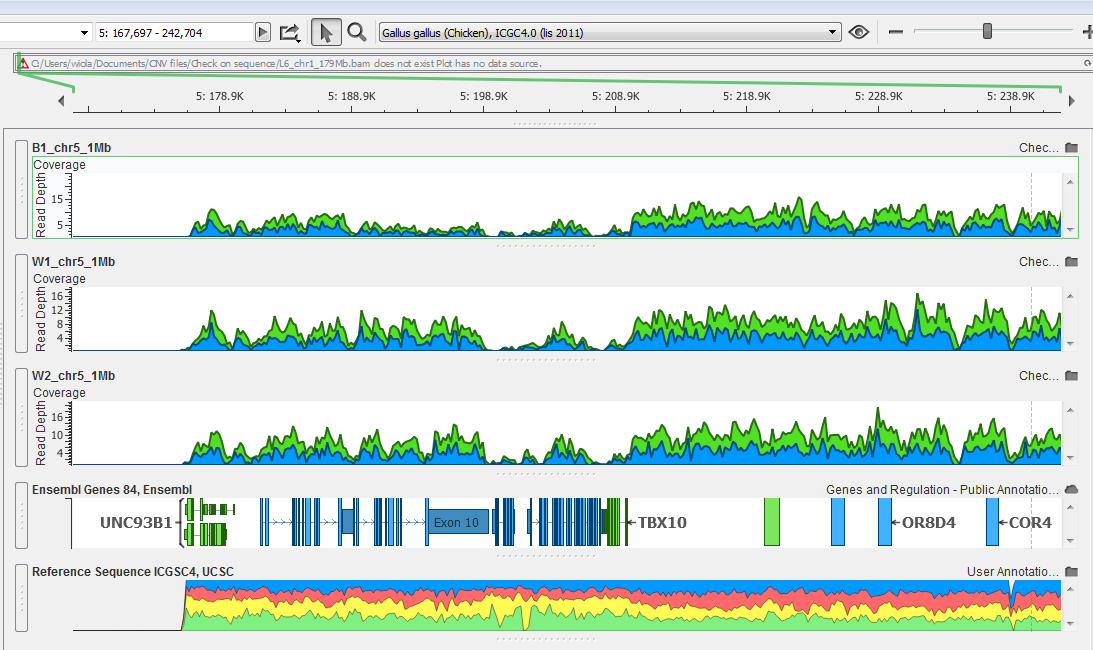


Figure S4. Complex CNV region on chromosome 5 of 0.1 Mb that segregates in line B1.


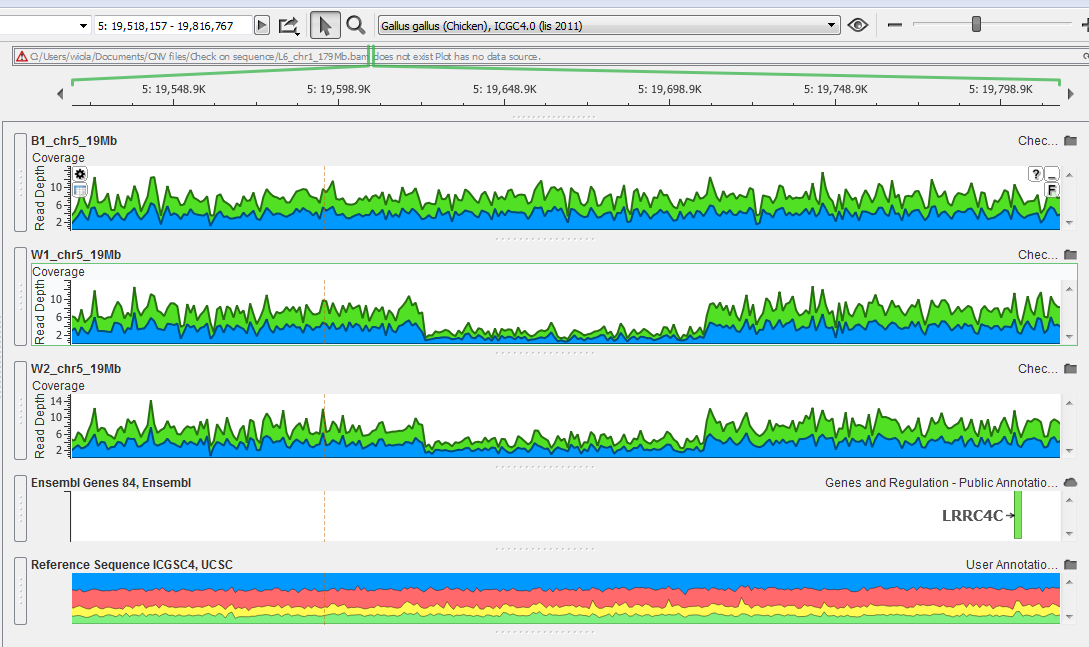


Figure S5. Deletion on chromosome 5 of 19.6 Mb that segregates within the white lines W1 and W2.


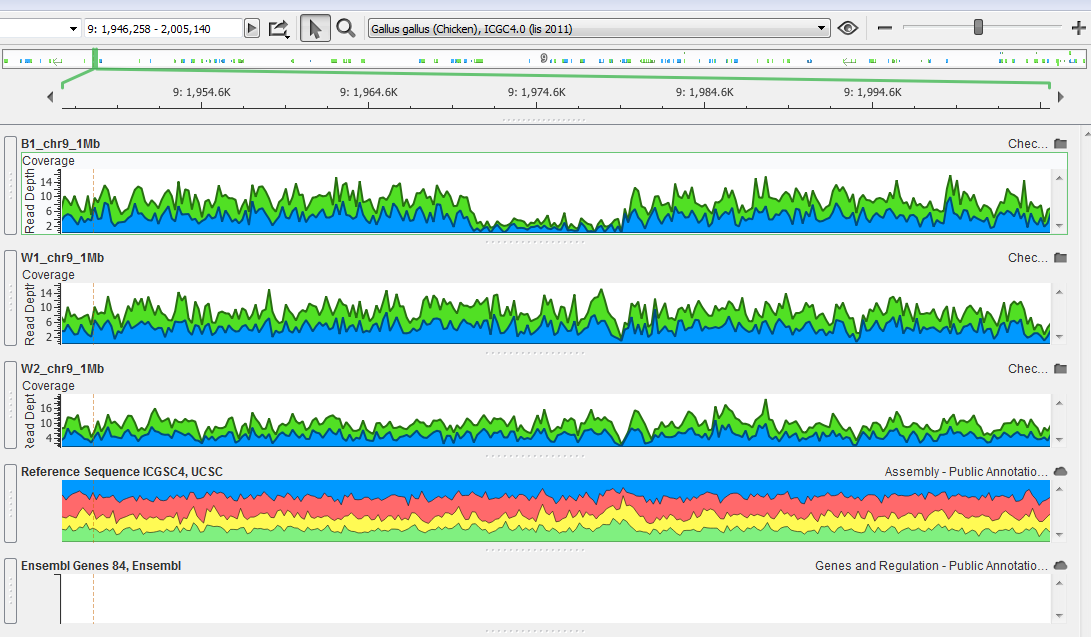


Figure S6. Deletion on chromosome 9 of 1.9 Mb that segregates within line B1.


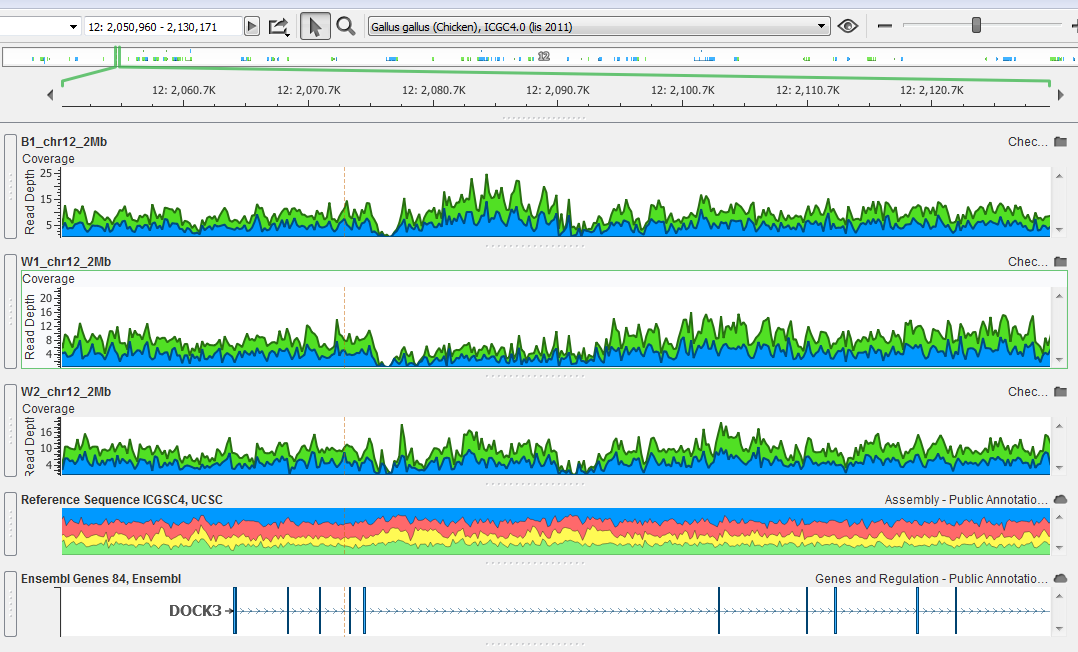


Figure S7. Complex CNV region on chromosome 12 of 2.0 Mb that overlaps with the DOCK3 gene and segregates within line B1 and W1. Duplication segregates in line B1 while deletion segregates in line W1.


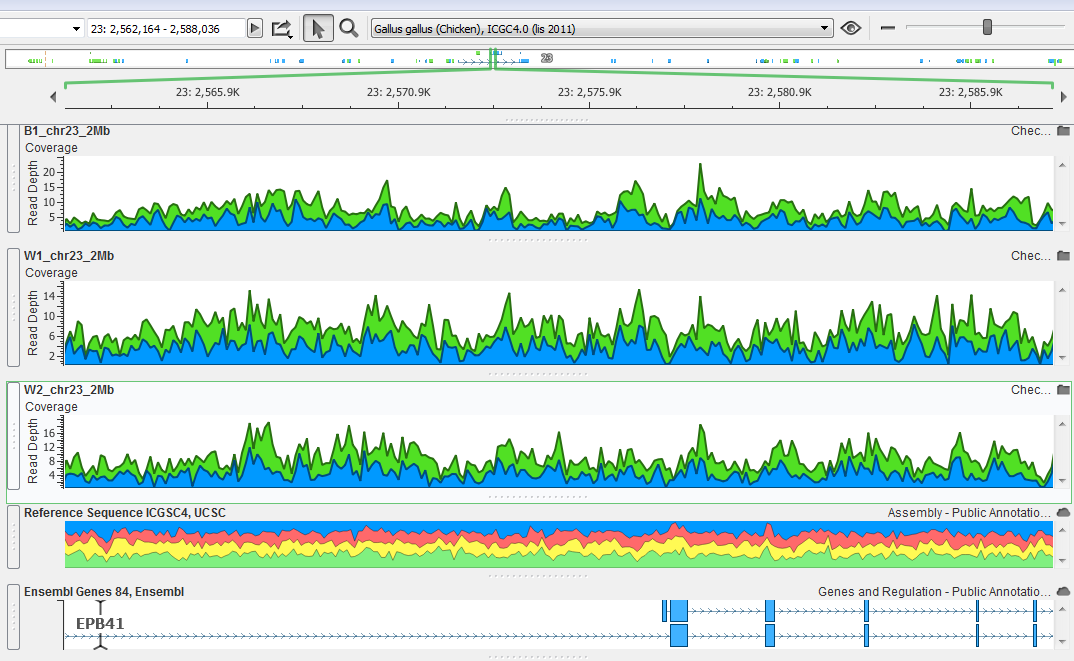


Figure S8. Deletion on chromosome 23 of 2.5 Mb that overlaps with the EPB41 gene and segregates within line B1.
